# Laparoscopic Management of Unruptured Cornual Ectopic Pregnancy in a 22-Year-Old Primigravida: A Case Report

**DOI:** 10.7759/cureus.56675

**Published:** 2024-03-21

**Authors:** Nadia Nishat, Aiysha Gul, Yoalkris E Salcedo, Ekta Chugh

**Affiliations:** 1 Family Medicine, Adichunchanagiri Institute of Medical Sciences, Mandya, IND; 2 Obstetrics and Gynecology, Mardan Medical Complex, Mardan, PAK; 3 Surgery, Universidad Iberoamericana, Santo Domingo, DOM; 4 Medicine, American University of Antigua, Osbourn, ATG

**Keywords:** primigravida mother, partial salpingectomy, patient outcomes, individualized management strategies, laparoscopic intervention, serum β-hcg, transvaginal ultrasonography, maternal mortality, interstitial ectopic pregnancy, cornual ectopic pregnancy

## Abstract

Cornual ectopic pregnancy, though rare, presents significant challenges in diagnosis and management. This case report details the clinical presentation and successful treatment of a 22-year-old primigravida experiencing symptoms of abdominal pain, nausea, and vomiting, ultimately diagnosed with an unruptured left cornual ectopic pregnancy. Employing a multidisciplinary approach involving clinical suspicion, beta-human chorionic gonadotropin (β-hCG) measurements, and transvaginal ultrasound findings, we underscored the importance of timely intervention to avert adverse outcomes. The patient underwent laparoscopic partial salpingectomy, resulting in minimal intraoperative blood loss and postoperative complications. Our experience highlights the effectiveness of laparoscopic intervention in managing cornual ectopic pregnancy and underscores the necessity of tailoring treatment strategies to individual patient circumstances. By adhering to established guidelines and advancing research efforts, we can further enhance outcomes for patients grappling with this challenging condition. This case emphasizes the critical role of early diagnosis, prompt intervention, and ongoing vigilance in the management of cornual ectopic pregnancies.

## Introduction

Cornual pregnancy, an uncommon occurrence representing around 2-4% of all ectopic pregnancies, occurs when a gestational sac implants and develops in the proximal and lateral regions of the uterus [1]. Diagnosis is based on clinical suspicion, measurement of beta-human chorionic gonadotropin (β-hCG), and observations from transvaginal ultrasound examinations, similar to other ectopic pregnancies [2]. This condition carries significant morbidity and mortality, highlighting the importance of prompt and accurate diagnosis, which directly influences treatment decisions and urgency [3].

Approaches to treatment range from conservative measures, such as administering methotrexate and adopting a watchful waiting approach, to more aggressive interventions like cornuotomy, cornual resection, or hysterectomy [4]. Hysteroscopy offers an additional option for effectively removing the gestational sac while minimizing disruption to uterine anatomy, although it often requires imaging guidance [5].

In this case report, we present the clinical scenario of a 22-year-old primigravida with 1.5 months of gestational amenorrhea, experiencing symptoms of abdominal pain, nausea, and vomiting. The subsequent evaluation confirmed a left cornual unruptured pregnancy, necessitating laparoscopic left partial salpingectomy.

## Case presentation

Upon arriving at our rural tertiary care hospital, a 22-year-old primigravida presented with persistent nausea, vomiting; dull and generalized abdominal pain with no radiation lasting for three days, accompanied by amenorrhea for the past one and a half months. She had no history of pelvic inflammatory disease or ureteric stones. Subsequent urine pregnancy testing confirmed her pregnancy. Clinical examination revealed mild pallor with no signs of abdominal tenderness, guarding, or rigidity. Additionally, per vaginal examination showed no distension in the pouch of Douglas or cervical motion tenderness.

**Figure 1:**
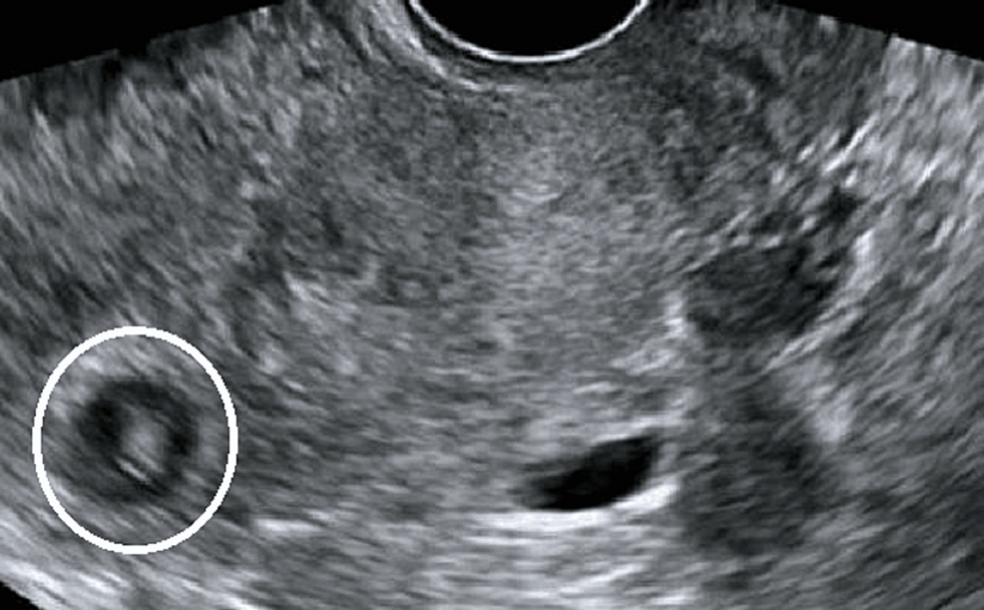
Transvaginal ultrasound shows left adnexal mass (shown by white circle)

Further investigation unveiled an elevation of beta-HCG levels (10500 mIU/ml) and mild anemia (Hb 11 gm/dl), while all other parameters remained normal. A transvaginal ultrasound revealed a heterogeneous mass lesion measuring 3 x 1.9 x 1.57 cm in the left adnexa (Figure 1), with no visible intrauterine gestational sac. Doppler ultrasound confirmed peripheral vascularity in the mass lesion (no image available), thus confirming the clinical suspicion of ectopic pregnancy.

**Figure 2:**
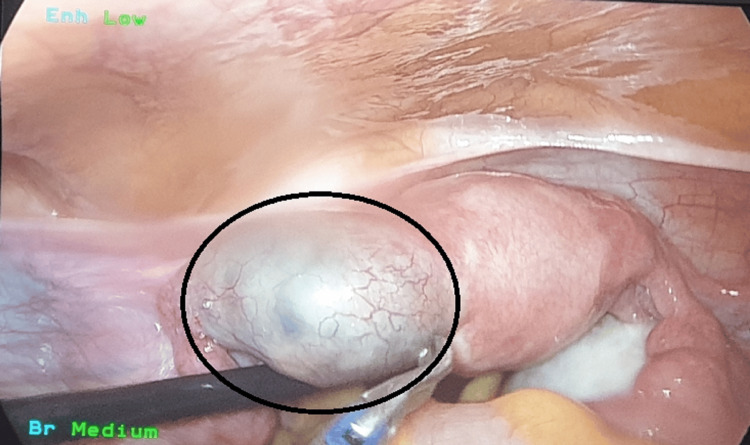
Laparoscopic view of left cornual unruptured ectopic pregnancy prior to partial salpingectomy (shown by black circle)

After confirming a definitive diagnosis of left tubal ectopic pregnancy, due to the patient's persistent symptoms and lack of improvement in laboratory parameters, The patient and her relatives were informed about the need for surgery. They were also informed about the possibility of a salpingectomy if the fallopian tube couldn't be saved. Subsequently, the patient underwent laparoscopy, during which an unruptured left cornual ectopic pregnancy was visualized (Figure 2).

**Figure 3:**
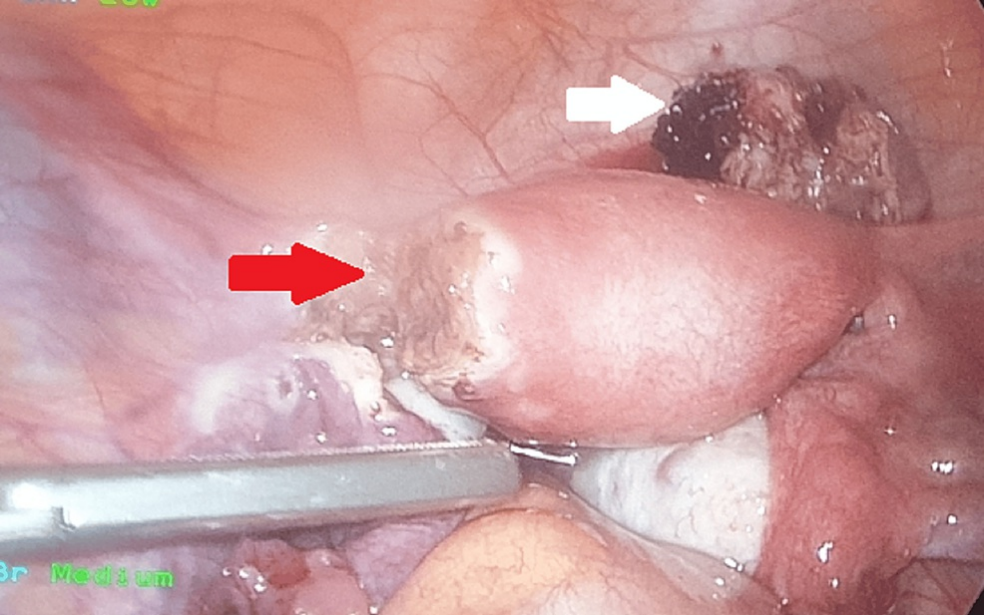
Laparoscopic view of left cornual ectopic pregnancy during partial salpingectomy. Post excision, the remaining part of the left tube (shown by red arrow) and the uterus are seen with the excised ectopic in the anterior uterovesical fold of the peritoneum (shown by white arrow)

During surgery, following informed consent from the husband, it was decided to proceed with a left partial salpingectomy, which was successfully completed with minimal blood loss (Figure 3). A repeat β-HCG on the third day postoperatively revealed a significant drop in levels, reaching 2345 mIU/ml. As there was a complete resolution of signs and symptoms, the patient was discharged on the fifth day. Subsequent follow-up after two weeks indicated that the patient was in good health, with normal transvaginal ultrasound findings and β-HCG levels.

## Discussion

Cornual pregnancy is rare (2-4% of ectopic pregnancies), with gestational sac implantation in proximal uterine regions. Diagnosis relies on clinical suspicion, β-HCG measurement, and transvaginal ultrasound observations, akin to other ectopic pregnancies [1,2]. The mortality rate linked to ectopic pregnancy can reach 9-14%, rendering it the leading cause of maternal mortality in the first trimester of pregnancy. Specifically, cornual pregnancy has been identified as a significant risk factor, with up to 48.6% of affected women experiencing uterine rupture between the sixth and 26th week of gestation [3].

Management strategies vary from conservative methods, such as administering methotrexate and expectant management, to more invasive interventions like cornuotomy, cornual resection, or hysterectomy [4]. Extensive case studies have confirmed the success of conservative treatment employing methotrexate, a study conducted on 20 cases of interstitial/cornual ectopic pregnancies revealed a 94% rate of successful pregnancy resolution. However, it is important to note this method is best suited for cases with lower levels of β-HCG [6]. Research with 42 women diagnosed with interstitial/cornual ectopic pregnancy revealed that lower initial levels of β-HCG were the sole statistically significant indicator of a favorable end result. In the successful group, the average β-HCG levels were measured to be 3216 mIU/ml [7]. The recent edition of the Royal College of Obstetricians and Gynaecologists guidelines also supports these findings [8]. In our case, the β-hCG levels reached a point where conservative management and observation were no longer considered safe. This prompted a preference for a more aggressive intervention.

The conventional approach to managing cornual ectopic pregnancy typically entails cornual resection, which can be performed via laparotomy or laparoscopy. However, in situations where life is threatened, hysterectomy may be considered as a final option [9]. In our case, we performed laparoscopic partial salpingectomy considering clinical conditions and recommendations.

The case highlights the challenges of cornual ectopic pregnancy, emphasizing the importance of timely diagnosis and tailored management. Laparoscopic partial salpingectomy emerged as a successful treatment option. Ongoing research and adherence to guidelines are vital for better outcomes. Our experience involved a clinically stable patient with an unruptured left cornual ectopic, managed effectively with minimal complications. Advanced imaging aids in early diagnosis, preventing severe complications.

## Conclusions

The presented case underscores the challenges and complexities associated with cornual ectopic pregnancy. Timely diagnosis and appropriate management are crucial for ensuring favorable outcomes for patients. Our case highlights the significance of individualized treatment approaches, with laparoscopic partial salpingectomy proving to be a viable option in addressing this condition. Continued research and adherence to clinical guidelines are essential for improving the management and outcomes of cornual ectopic pregnancies. We encountered a case involving a clinically stable patient with an unruptured left cornual ectopic, which we managed successfully with laparoscopic partial left salpingectomy, resulting in minimal intraoperative blood loss and postoperative complications. With the advancement of imaging modalities, unruptured ectopic pregnancies are increasingly being diagnosed earlier in pregnancy, facilitating prompt management to prevent catastrophic bleeding and mortality.
